# Early-stage reconstruction of flexor pollicis longus using palmaris longus in a patient with compartment syndrome of forearm: a case report

**DOI:** 10.1186/s13256-022-03530-4

**Published:** 2022-08-25

**Authors:** Muneyoshi Fujita, Hideaki Miyamoto, Taketo Kurozumi

**Affiliations:** Trauma and Reconstruction Center, Teikyo University Hospital, 2-11-1 Kaga, Itabashi-ku, Tokyo, 173-8606 Japan

**Keywords:** Case report, Compartment syndrome, Flexor pollicis longus reconstruction, Palmaris longus, Tendon transfer

## Abstract

**Background:**

The flexor pollicis longus is the most vulnerable muscle in acute compartment syndrome of the forearm. Reconstruction of a dysfunctional flexor pollicis longus is occasionally necessary following compartment syndrome of the forearm.

**Case presentation:**

A 42-year-old Japanese man injured his left forearm in a motor vehicle accident. Open radial shaft fracture and acute compartment syndrome of the left forearm was diagnosed. We performed a fascial release of the forearm and debridement of the involved myonecrosis of the flexor pollicis longus. At second-look operation (3 days after the initial release), we performed palmaris longus tendon transfer to the flexor pollicis longus tendon. At 6-month follow-up, the patient had no complaints and returned to his job. At 2-year follow-up, the patient had achieved 88% of pinch strength, compared with the contralateral hand, and scored 11.4 on the QuickDASH score.

**Conclusions:**

Palmaris longus transfer performed immediately after injury is simple and does not require an additional surgical approach. Hence, early palmaris longus tendon transfer, which can provide satisfactory outcomes, could be considered as a potential choice for flexor pollicis longus reconstruction in patients with compartment syndrome of the forearm.

## Background

Acute compartment syndrome can lead to advanced myonecrosis in the forearm. Commonly, the volar muscles are the most severely affected in compartment syndrome, followed by the muscles of the dorsal compartment and the lateral compartment. In particular, the flexor digitorum profundus (FDP) and flexor pollicis longus (FPL), included in the deep muscles, are the most vulnerable in compartment syndrome [1]. Therefore, reconstruction of dysfunctional flexor tendons is occasionally necessary following compartment syndrome. In this report, we introduce the technique of early palmaris longus (PL) tendon transfer for FPL reconstruction and describe the surgical outcomes in a patient with compartment syndrome treated using early PL tendon transfer.

## Case presentation

**Fig. 1:**
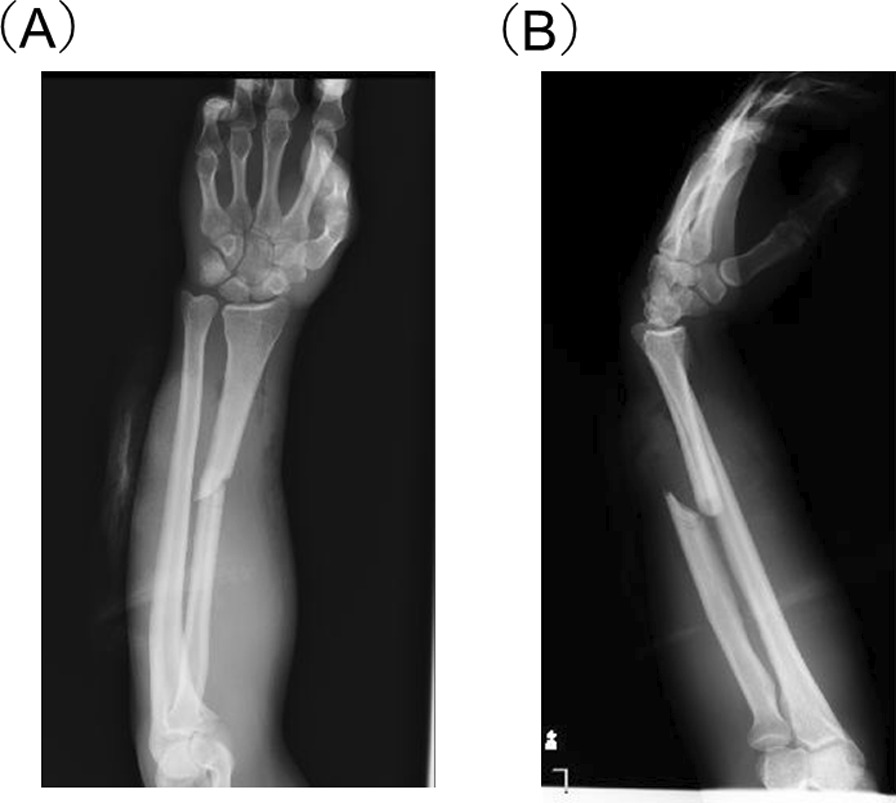
Conventional radiograph showing isolated radial shaft fracture. **A** Anteroposterior view, **B** lateral view

**Fig. 2:**
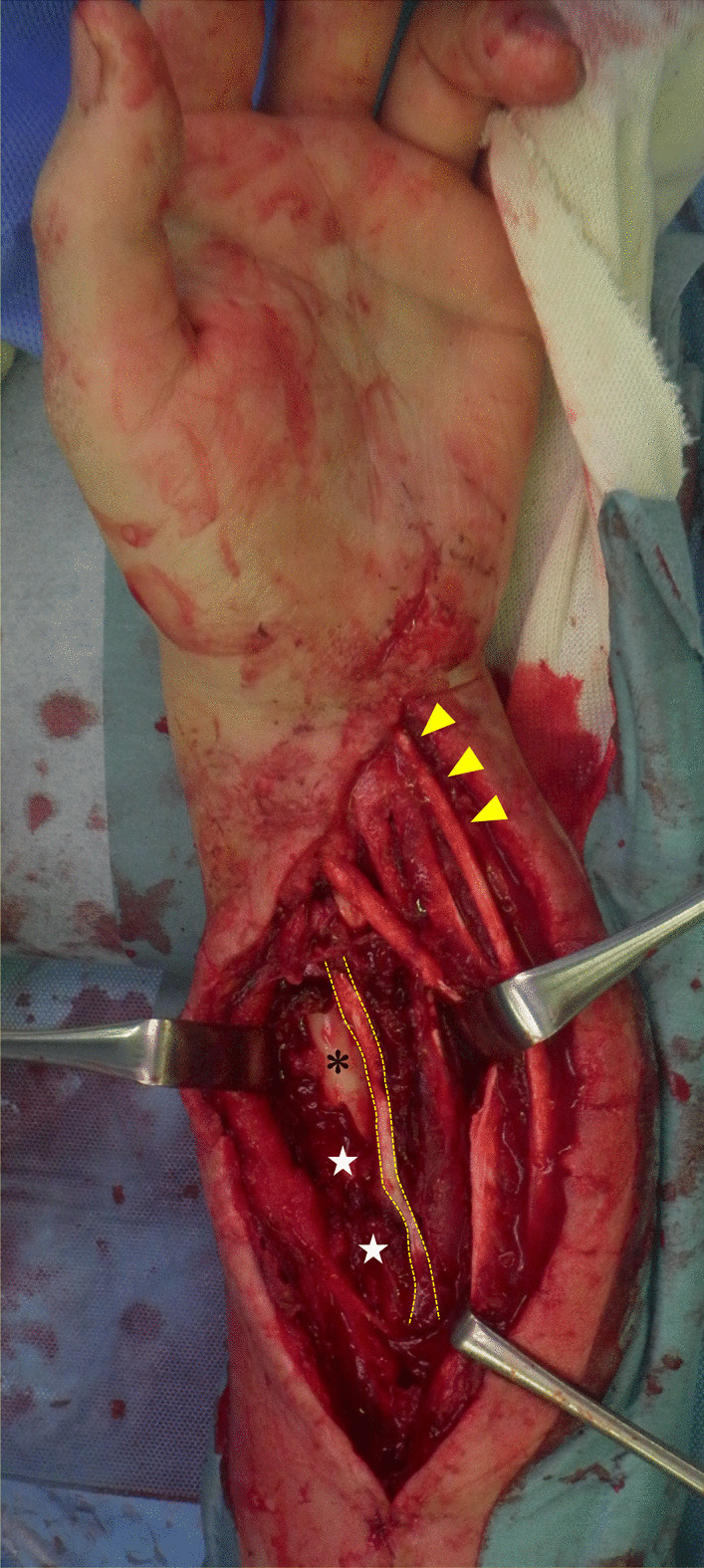
Operative findings of the forearm. Complete rupture of the pronator quadratus muscle and partial myonecrosis of the FPL and FDP at the muscle belly. Yellow arrowheads, PL tendon; asterisk, radius; white stars, debrided muscle necrosis of the pronator quadratus and the FDP; yellow dots, debrided FPL with functional loss

A 42-year-old Japanese man presented to our emergency department immediately after his motorcycle collision. The patient had a history of alcohol and tobacco use but otherwise no significant medical history. His left forearm was caught between the motor vehicle and the road, and became highly compressed and retracted. The patient was hemodynamically stable with a Glasgow Coma Scale of 15/15, a blood pressure of 120/60 mmHg, pulse rate of 90 beats per minute, respiratory rate of 24 breaths per minute, body temperature of 36.1 °C, and oxygen saturation of 99% on room air. Laboratory test results were as follows: hemoglobin 16.3 g/dL; hematocrit 46%; white blood cells 8100/mm^3^, with no left shift; platelets 195,000/mm^3^; urea 14.5 mg/dL; creatinine 0.8 mg/dL; total bilirubin 1.0 mg/dL; direct bilirubin 0.07 mg/dL. Major vascular and visceral injuries were not suspected. Physical examination and radiography showed an open radial shaft fracture with marked swelling of the left forearm (Fig. 1). The patient was given 0.5 mL of tetanus toxoid vaccine intramuscularly and administered prophylactic antibiotic using intravenous piperacillin/tazobactam 4.5 g. Compartment pressure was measured in three different areas of the forearm, showing 40 mmHg in the volar component and 30 mmHg in the dorsal forearm compartment. Acute compartment syndrome was diagnosed on the basis of the clinical symptoms and compartment pressures of the forearm. Five hours after the injury, surgery was performed under general anesthesia. First, we made a curvilinear skin incision on the volar aspect of the forearm from the biceps tendon to the palm of the hand for carpal tunnel release (Fig. 2). We performed a debridement of the involved muscles; however, we could not perform an internal fixation or immediate reconstruction owing to the risk of advancing the myonecrosis of the forearm muscles. After release of the superficial and deep flexor compartments, pressure in the dorsal compartment decreased. The wound was closed with vessel tape, utilizing the shoelace technique. After the release, the patient was not able to flex the interphalangeal joint of the thumb. At second-look operation (3 days after the initial release), swelling of the left forearm had reduced with no evidence of infection. Therefore, we performed open reduction and internal fixation for the left radius fracture and PL tendon transfer to the FPL tendon. Intraoperatively, the presence of the intact PL tendon was confirmed. The PL tendon was advanced to the FPL tendon, and an interlacing suture was performed thrice. The tendon was transferred with the wrist in 30° of dorsiflexion and the metacarpophalangeal and interphalangeal joints of the thumb in 45° of flexion. The wrist and thumb were immobilized in a slightly flexed position using a splint for 3 weeks, following which active flexion and extension exercises of the thumb were started. The patient was discharged 32 days after hospital admission in a relatively good state of health. Six weeks after the reconstruction, passive extension exercises of the thumb were started. At 6-month follow-up, the patient had no complaints and had returned to his former job, despite slight residual muscle weakness of the FPL. The patient regained the full range of thumb interphalangeal joint flexion–extension. At 2-year follow-up, the patient had acquired 88% of pinch strength compared with the contralateral hand and scored 11.4 on QuickDASH score.

## Discussion and conclusions

Here we describe our experience in treating FPL reconstruction using PL tendon transfer at an early stage of compartment syndrome of the forearm with satisfactory result.

When performing a tendon transfer, there are several considerations in the selection of the donor muscle. First, the donor muscle should be nonparalytic. Second, the transfer must run in a straight line from the donor muscle’s origin to the insertion of the tendon it is to drive. Acute angulation of the transfer should be avoided at the pulley. Third, the surgeon must be aware of the amplitude of the tendon excursion for each muscle. Fourth, the muscle chosen as the donor for transfer must be sufficiently strong to perform its new function in its altered position [2]. There is no literature that shows that brachioradialis (BR) muscle transfer to the FPL yields poor results. However, several studies [3–5] have shown that postoperative pinch strength is reduced because the transferred BR continues to produce an elbow flexor moment because of its proximal attachment on the humerus. A strong contraction of the BR in pinch must be balanced by a strong contraction of the elbow extensor.

In case of compartment syndrome, we considered the PL to be more useful than the BR for early reconstruction of the FPL, because the PL is spared. Moreover, the PL, which runs parallel to the FPL tendon, is easily accessible in the surgical field after fascial release of the forearm. The amplitude of the PL tendon excursion is 40 mm, which is comparable to the 50 mm excursion of the FPL tendon [6]. In contrast, the BR has poor excursion because of the tight connection of its tendon to the antebrachial fascia. Extensive dissection of the BR from its surrounding facial attachments is required. Koh *et al*. described successful outcomes following PL tendon transfer for FPL reconstruction in three cases of anterior interosseous nerve syndrome [7]. They showed that PL transfer resulted in an average pinch strength of 90%, compared with the contralateral hand, in their patients. Similarly, in our case, the patient had obtained satisfactory clinical outcomes, including an average pinch strength and few limitations in functional performance.

Early intervention with tendon transfer is usually not warranted in areas with open wounds; nevertheless, early PL transfer soon after injury is a simple procedure that does not require an additional surgical approach. Even if the transferred PL tendon does not regain a sufficient amount of function, other tendons can be used as salvage donors, with minimal impairment of hand function. We emphasize that early tendon transfer with PL can provide satisfactory results, and could be considered as a potential choice for FPL reconstruction following compartment syndrome of the forearm.

## Data Availability

Not applicable.

## Abbreviations

BR: Brachioradialis
FDP: Flexor digitorum profundus
FPL: Flexor pollicis longus
PL: Palmaris longus
